# Restless Legs Syndrome: Known Knowns and Known Unknowns

**DOI:** 10.3390/brainsci12010118

**Published:** 2022-01-16

**Authors:** Elena Antelmi, Lorenzo Rocchi, Anna Latorre, Daniele Belvisi, Francesca Magrinelli, Kailash P. Bhatia, Michele Tinazzi

**Affiliations:** 1 Neurology Unit, Parkinson Disease and Movement Disorders Division, Department of Neurosciences, Biomedicine and Movement Sciences, University of Verona, 37134 Verona, Italy; elenaantelmi@gmail.com (E.A.); f.magrinelli@ucl.ac.uk (F.M.); michele.tinazzi@univr.it (M.T.); 2 Department of Clinical and Movement Neurosciences, UCL Queen Square Institute of Neurology, University College London, London WC1N 3BG, UK; a.latorre@ucl.ac.uk (A.L.); k.bhatia@ucl.ac.uk (K.P.B.); 3 Department of Medical Sciences and Public Health, University of Cagliari, 09124 Cagliari, Italy; 4 Department of Human Neuroscience, Sapienza University of Rome, 00185 Rome, Italy; daniele.belvisi@uniroma1.it; 5 IRCCS Neuromed, 86077 Pozzilli, Italy

**Keywords:** restless legs syndrome, sleep-related movement disorder, sensorimotor interaction, circadian disorders, dopamine, iron, sensory gating

## Abstract

Although restless legs syndrome (RLS) is a common neurological disorder, it remains poorly understood from both clinical and pathophysiological perspectives. RLS is classified among sleep-related movement disorders, namely, conditions characterized by simple, often stereotyped movements occurring during sleep. However, several clinical, neurophysiological and neuroimaging observations question this view. The aim of the present review is to summarize and query some of the current concepts (known knowns) and to identify open questions (known unknowns) on RLS pathophysiology. Based on several lines of evidence, we propose that RLS should be viewed as a disorder of sensorimotor interaction with a typical circadian pattern of occurrence, possibly arising from neurochemical dysfunction and abnormal excitability in different brain structures.

## 1. Introduction

Restless legs syndrome (RLS) is a common neurological disorder affecting up to 15% of the general population [1,2,3]. It is categorized as a sleep-related movement disorder (MD) due to its peculiar occurrence, usually during or shortly before sleep [4]. RLS has a significant impact on daily activities and deeply affects patients’ quality of life [5].

The diagnosis is clinical and is based on five criteria: (1) an urge to move the legs, often associated with unpleasant leg sensations; (2) induction or exacerbation of symptoms by rest; (3) symptom relief upon activity; (4) daily fluctuations of symptoms with worsening in the evening and at night; (5) exclusion of other medical and behavioral conditions that can mimic RLS [4,5,6,7]. Although RLS predominantly affects the legs, other body parts can be involved, including the arms, abdomen, pelvis, and even the bladder and face [6].

RLS is classified as primary (idiopathic, iRLS) or associated with other medical conditions [8]. The former often shows an autosomal dominant transmission and has specific genetic risk factors, whereas medical conditions more frequently associated with RLS include iron deficiency, end-stage renal disease, pregnancy, peripheral neuropathy, diabetes mellitus, and Parkinson’s disease [3,9]. Effective medications include dopamine agonists (pramipexole, ropinirole, rotigotine), levodopa, α2δ agonists (gabapentin, pregabalin), iron supplementation and opioids [10,11,12].

The pathophysiology of iRLS remains controversial [13]. Dopaminergic dysfunction has been suggested on the basis of clinical improvements observed after dopaminergic treatment. Post-mortem, neuroimaging and biohumoral studies have suggested that brain iron deficiency (BID) may also play a role in RLS pathophysiology [14]. More recently, the involvement of other neurotransmitter pathway dysfunctions, including GABA and adenosine, has been suggested [14]. Structural [15] and functional [16] MRI studies performed in iRLS patients during symptom expression have demonstrated abnormal activation of several brain areas, including the cerebellum, contralateral thalamus, midbrain, dorsolateral prefrontal cortex, anterior cingulate cortex, pre- and post-central gyri and putamen, suggesting multisystem involvement [17,18,19].

RLS is classified as a sleep-related movement disorder (SRMD), according to the third edition of the International Classification of Sleep Disorders. Despite this, and notwithstanding the comorbidity between RLS and other SRMD (such as periodic limb movement disorder, PLMD), several clinical, neurophysiological and neuroimaging observations question this view [20,21,22].

The aim of the present review is to summarize and query some of the current concepts of RLS pathophysiology (known knowns) and to identify open questions (known unknowns), which might drive future research and shed light on this disorder.

## 2. Why RLS Is Not a Sleep-Related Movement Disorder

RLS is classified among sleep-related MDs, namely, conditions characterized by simple, often stereotyped movements occurring during sleep [4]. Indeed, such conditions, such as PLMD, can present together with RLS. However, this definition appears to be misleading for two main reasons. First, RLS has a circadian pattern of occurrence, but symptoms are not restricted to sleep time and can occur during rest (e.g., while watching television, at the cinema or during long flights), even during the daytime, in up to 60% of patients [23]. Moreover, although RLS can operate as a counter to sleep homeostatic drive, night disruption due to RLS symptoms is not a mandatory diagnostic criterion. Secondly, although movements in MDs are involuntary by definition, those in RLS are under voluntary control and are performed with the intent to relieve discomfort. Indeed, the main complaint by RLS patients is an uncomfortable sensation in the legs, associated with a sense of internal restlessness and urge to move them, which is relieved by voluntary leg movements. Considering this, RLS may share similarities with tics or akathisia [24,25]. However, differently from these disorders, somatosensory symptoms in RLS (variously described as an awkward sensation, i.e., ”crawling”, “tingling”, “restlessness”, “cramping”, “creeping”, “pulling”, “pain”, “electric shocks”, “tension”, “itching”, “burning” and “pricking”) are reported as spontaneous by up to 80% of RLS patients and differ from the inner feeling of compulsion to move, typical of akathisia and tics.

The most common strategy to alleviate the discomfort caused by RLS is moving the legs. Interestingly, however, symptom reduction seems to be correlated with the degree of alertness produced by movement, more than with movement intensity [7]. Other strategies for symptom relief usually involve a degree of somatosensory input, such as massaging the legs, tying a cloth/rope tightly around them, dipping the feet in cold water, or applying ice over calves [7]. For these reasons, idiopathic RLS may be considered a primary sensory disorder, which triggers a voluntary motor response. This might be in keeping with sensory neuropathies [8] and spinal cord lesions [26] being among the causes of secondary RLS, possibly due to altered sensory inputs or sensorimotor interactions at the spinal cord level, which has been suggested in RLS [27]. What remains a purely motor phenomenon in the context of RLS are PLMDs, which occur in nearly 85% of RLS patients [4]. Considering the strong association between PLMD and RLS, the former is often considered a supportive criterion for the diagnosis of RLS, although it is neither necessary nor sufficient for it. PLMD, however, is also very common in patients without RLS, being found in 7.6–25% of the general population and between one-third and two-thirds of older adults [28,29].

## 3. Why RLS Should Be Considered a Disorder of Sensorimotor Interaction, Rather than a Motor Disorder

Growing evidence supports the view of RLS as a derangement of sensorimotor interaction. Psychophysical studies in iRLS have showed a reduced pain threshold and increased somatosensory threshold, without evidence of small fiber neuropathy [30]. On the other hand, neurophysiological studies have demonstrated increased excitability of the primary somatosensory cortex, with impairment of the somatosensory gating control [21], whereas brain MRI scans have revealed morphologic changes in the primary somatosensory system, with decreased cortical thickness in the bilateral postcentral gyrus [20]. By using a multimodal MRI approach, Stefani and colleagues showed that progressive white matter impairment of somatosensory circuits in iRLS was associated with the perception of sensory discomfort in the legs [15]. A central origin of the symptoms has therefore been hypothesized, likely due to a decreased supraspinal inhibitory influence on the spinal cord, resulting in increased spinal excitability [22].

Two mechanisms that may have an important role in altered somatosensory processing in RLS are BID and dopaminergic dysfunction. BID, especially in the substantia nigra (SN), with or without systemic iron deficiency, is considered an ubiquitous finding in RLS patients, with neuroimaging, neurochemical and histological evidence from humans and animal models [31,32,33,34,35,36,37,38]. RLS has been associated with low cerebrospinal fluid (CSF) ferritin and high CSF transferrin levels [34,35]; imaging studies have revealed decreased iron concentrations in the SN, red nucleus, thalamus and striatum [36]. Similar evidence has arisen from post-mortem studies [37] and animal models [33]. Further supporting a possible role of BID in RLS pathophysiology is the evidence that BTBD9 and MEIS1, two genetic risk loci for RLS, are involved in brain iron metabolism [9]. Interestingly, BID has been implicated not only in the modulation of motor activity and alertness, but also sensory perception: iron-deficient rats exhibit lower pain thresholds during dark periods and increased acute and chronic pain responses [38].

Experimental and clinical evidence suggests that dopamine also plays a role in sensory and pain perception and in modulating central nervous system (CNS) excitability. Moreover, basal ganglia, one of the main targets of central dopaminergic innervation, are involved in the sensory-discriminative dimension of pain and provide sensory gating of nociceptive information to motor cortical areas [39].

Clinically, RLS appears to result, at least in part, from decreased dopaminergic signaling, as suggested by the evidence that RLS symptoms improve when increasing dopaminergic stimulation through dopamine receptor agonists, whereas they worsen with antidopaminergic medications. However, it has been suggested that phenotypic manifestations reflect decreased post-synaptic dopamine signaling and pre-synaptic hyperdopaminergic states [40,41,42], with neuroimaging studies showing evidence of increased tyrosine hydroxylase activity and dopamine synthesis, decreased dopamine transporter activity, and decreased dopamine reuptake [31]. Accordingly, resting-state functional MRI studies have shown that functional connectivity within the dopaminergic networks, including the nigrostriatal, mesolimbic, and mesocortical circuits, was lower in patients with RLS than in controls [43].

The involvement of neurotransmitters other than dopamine has also been reported. For instance, a hypoadenosinergic state, potentially linked to BID, may lead both to hyperglutamatergic and hyperdopaminergic states [44]. The latter might also be directly linked to BID [31]. Furthermore, endogenous opioids have been found to be decreased in sensory, but not in motor pathways, in RLS patients [45]. Therefore, the hyperdopaminergic state in RLS may lead to a decrease in endogenous opiates, which may contribute to the abnormal processing of sensory stimuli [46].

The hypothesis that RLS may represent a network disorder, rather than a purely dopaminergic one, is also supported by recent evidence coming from functional connectivity studies. Tuovinen and coworkers reported that patients with RLS have increased connectivity within salience and executive networks and a reduced cerebello-frontal connectivity [47]. The observation that RLS is a multipathway network disorder has relevant therapeutic implications and suggests the possibility to identify new non-dopaminergic therapeutic targets.

## 4. Why RLS Is a Circadian Disorder, Rather than a Sleep-Related Disorder

The occurrence of RLS symptoms shows a strong circadian pattern, with a peak in the early portion of the sleep period (23:00–04:00 h) and a nadir during the early portion of the wake period (09:00–14:00 h). Although the function of the central circadian pacemaker does not seem to be abnormal, the severity of symptoms might be indirectly modulated by some factors undergoing circadian variation. Human data show a circadian variation in dopamine release, with a pattern characterized by an increase in the morning and a nadir in the late evening/night [48]. This is possibly due to a dopamine–melatonin interaction, because the peak of melatonin coincides with the nadir of dopamine present in the striatum [48]. From an evolutionary perspective, this could be explained by the lower need for dopamine at night, when movement is not required.

It might be supposed that the hyperdopaminergic state, and consequent downregulation of dopamine reuptake, reaches a critical point at evening–night-time, when the release of additional neurotransmitters determines a drop in dopamine release. Post-synaptic downregulation of D2 receptors due to a hyperdopaminergic state may result in low dopaminergic signaling when dopamine levels are physiologically low, i.e., in the evening, leading to a relative night-time dopamine activity deficit and the occurrence of RLS symptoms. This may explain why dopaminergic agents given at night are effective treatment options.

BID, dopamine and melatonin levels seem to be closely related [48]: serum iron shows a marked circadian variation, with a nadir in the evening and early night [49], time which coincides with the maximal severity of RLS symptoms. Thus, in general, circadian variation in serum iron seems to mirror the circadian variation in CSF dopamine. It should be noted, however, that in some RLS patients, the correlation between serum and CSF ferritin might be weak [50].

The dopaminergic model could also explain the so-called augmentation, a unique iatrogenic phenomenon affecting up to 70% of RLS patients under long-term dopaminergic treatment. This consists of an earlier onset of RLS symptoms in the day, a shorter latency of symptoms at rest, a spread of symptoms to other parts of the body, or a greater intensity of symptoms [3,4]. Another key feature of augmentation is the paradoxical effect of treatment: a dose increase causes symptom worsening, whereas a reduction leads to improvement. Dopaminergic treatment can therefore trigger a phase advance of circadian rhythm, expressed as an anticipation of dim light melatonin, a further drop in dopamine and the anticipation of RLS symptoms [13].

However, data on neurotransmitter releases in RLS are controversial. Some studies have reported no differences in serotonin and dopamine CNS levels in RLS versus controls at evening time [51], whereas others have identified higher concentrations of dopamine metabolites in RLS in the evening versus morning compared with healthy controls, with reduced ferritin levels [40].

Nevertheless, it is important to highlight that, although this evidence suggests that RLS is a circadian rather than a sleep disorder, it is often related to PLMD, which is the primary sleep disturbance of RLS and is associated with (and presumably causes) poor sleep quality in these patients.

## 5. Conclusions and Future Research Perspectives

RLS is a puzzling disorder that remains poorly understood from both clinical and pathophysiological perspectives. Recent studies have suggested that RLS should be contemplated as a complex network disorder related to several neurochemical dysfunctions, with a typical circadian pattern of occurrence and evidence of abnormal excitability within the CNS. Neurophysiological studies in humans [52] and animal models [53] point toward a hyperfunctioning of excitatory mechanisms, rather than hypofunctioning of inhibitory mechanisms, including increased activities in the D1R pathway and hyperexcitability of motor circuitry [52]. The somatosensory system seems to play an important role, and it has been proposed that the abnormal processing of afferent inputs may be involved in releasing otherwise unnecessary motor outputs [54].

We might hypothesize that abnormal sensorimotor processing, related to a hyperdopaminergic/hyperglutamatergic state and to hypoadenosinergic state, triggered by BID, can be temporally reset by somatosensory input acting through a gating mechanism, as suggested for other neurological disorders, such as dystonia or tics [24]. Intrinsic factors (i.e., active movement, pressure, or alertness) are well known to play a pivotal role in modulating RLS symptoms, although little is known about extrinsic factors (i.e., visual, auditory or somatosensory stimuli), which might be functionally relevant as well.

**Figure 1:**
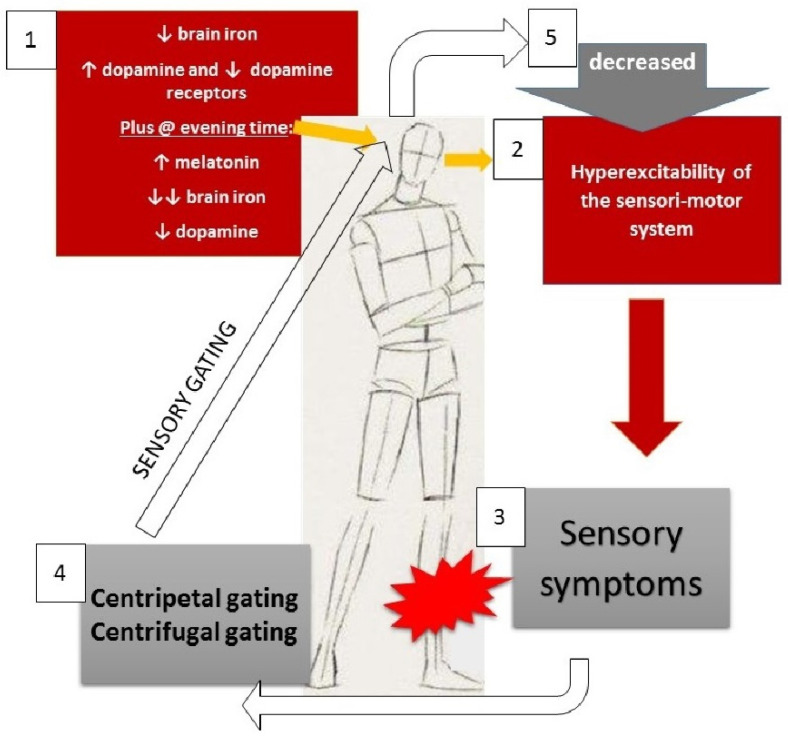
Proposed pathophysiological model for RLS. Centripetal gating is achieved by passive movements, massage, pressure, cold water, streaking, etc.; centrifugal gating is obtained by active movements. Arrows refer to increase/decrease of the mentioned compounds (“↑”: increase; “↓”: decrease; “↓↓”: substantial/consistent decrease.

The circadian occurrence of neurochemical abnormalities might be related to the fact that these are at subthreshold levels until additional neurochemicals (i.e., melatonin and potentially others) or other unknown factors determine the reach of a critical level (Figure 1). However, the interplay between all these intrinsic and extrinsic factors is not completely understood, and there are many knowledge gaps to be filled in order to better understand the nature of the disease.

In conclusion, we propose that RLS should be viewed as a circadian sensorimotor disorder, rather than a sleep-related MD. Future research should focus on improving the clinical characterization of extrinsic and intrinsic modulators of RLS symptoms and on investigating their effect on sensorimotor cortex excitability and integration. Further studies are also needed to disentangle the link between RLS symptoms and compounds showing circadian variations in their CSF concentration (e.g., ferritin, transferrin, dopamine, hypocretin-1, homovanillic acid, 5-Hydroxyindoleacetic acid) [14] and develop an integrated model of the disease, which can ultimately assist in the development of new treatment avenues. In this regard, promising targets might be represented by the glutamate and adenosine neurotransmission systems, whose modulation has been suggested to be effective in alleviating RLS symptoms [55].

## Data Availability

Not applicable.
